# Functional Response and Predation Potential of *Carabus elysii* Adults against the Terrestrial Slug *Agriolimax agrestis*

**DOI:** 10.3390/insects12121135

**Published:** 2021-12-18

**Authors:** Lin Jiang, Runa Zhao, Hui Tian, Xuesan Wu, Feng Guo, Wenlong Chen

**Affiliations:** 1 Provincial Key Laboratory for Agricultural Pest Management of Mountainous Region, Institute of Entomology, Guizhou University, Guiyang 550025, China; jianglin0095@163.com (L.J.); zhao21373@163.com (R.Z.); gzdxTH2021@163.com (H.T.); 2 Agricultural Integrated Service Centre, Xinbao Buyi Township, Wudang District, Guiyang 550025, China; xswu1994@163.com; 3 Agricultural and Rural Service Center, Longping Town, Bozhou District, Zunyi 563129, China; gf199305@163.com

**Keywords:** *Carabus elysii*, *Agriolimax agrestis*, predation, intraspecific interference

## Abstract

**Simple Summary:**

*Agriolimax agrestis* is one of the most important cash crop pests in China, widely affecting tobacco, vegetables, edible mushrooms, and other crops. Not only does it cause direct damage to plants but it also spreads bacteria that are harmful to plants and humans. Currently, the control of *A. agrestis* relies mainly on chemical agents; however, the heavy application of chemicals often leads to ecological damage. In this study, using *Carabus elysii* as a natural enemy, the predatory ability of adult *C. elysii* on *A. agrestis* was assessed under indoor conditions for the first time. The results show that *C. elysii* adults have a strong predatory ability on different sizes of slugs, especially juvenile slugs, and the female adults have a better predatory ability than the male adults. In general, *C. elysii* has strong potential to control *A. agrestis* and can be used as an effective control measure.

**Abstract:**

Terrestrial slugs are a prominent agricultural pest worldwide. To mitigate the negative effects of chemical pest control, biological control involves the use of natural enemies to reduce the impact of target pests. Numerous insects are natural predators of slugs. This study evaluated potential of the predatory species, *Carabus elysii* Thomson (Coleoptera: Carabidae) to biologically control the terrestrial slug, *Agriolimax* *agrestis*. Laboratory experiments were conducted to investigate the functional response, searching efficiency, and interference effect of female and male *C. elysii* adults regarding adult, immature, and juvenile *A. agrestis* individuals. The results show that both female and male ground beetle adults are functionally capable of preying on different sizes of terrestrial slugs. *C. elysii* exhibited Holling type II functional responses when preying on *A. agrestis*. The maximum daily prey consumption was 35.5 juveniles, 25.1 immatures, and 17.1 adults for adult females and 26.9 juveniles, 20.3 immatures, and 11.6 adults for adult males. The searching efficiency of female *C. elysii* adults regarding *A. agrestis* was always higher than that of male adults for identical ages and densities of *A. agrestis.* Moreover, the predation of *C. elysii* on slugs was affected by predator density. The disturbance coefficient of male *C. elysii* were the highest on adult *A. agrestis*. The results of this study suggest that female *C. elysii* exhibit a high potential for the biological control of *A. agrestis*.

## 1. Introduction

Slugs (Mollusca: Gastropoda: Stylommatophora) are prominent agricultural and horticultural pests in temperate and tropical regions worldwide. In addition to feeding on all parts of plants, they are vectors for numerous plant pathogens that can reduce both the aesthetic appearance of plants and crop yields [1,2]. Furthermore, they disseminate parasites that can be harmful for humans, domestic animals, and wild mammals. Slugs of this order are widely distributed in all provinces and regions of China [3,4]. Various species of slug (e.g., *Agriolimax agrestis* Linnaeus, *Phiolomycus bilineatus* Bonson, and *Limax flavus* Linnaeus) occur frequently in southern China and are an agricultural pest affecting vegetables, tobacco, and other crops [4,5,6]. Among them, the terrestrial slug *A. agrestis* is a very common and harmful mollusk and is distributed worldwide. The slugs are hermaphrodites with male and female reproductive organs and can fertilize themselves or mate [7,8]. The activities of slugs have been governed not by any definite annual cycle but by the prevailing condition of the weather [5,9]. In the Guiyang area (SW China), the slugs can be found all year round and have become the most serious and economically damaging pest in spring and summer [5]. *A. agrestis* occurs in two to six generations a year, with complex overlapping generations. The period of occurrence varies between generations, ranging from 9 to 14 months (270~420 d) [10,11,12]. Adults can lay eggs 2–3 d after mating, and the egg period is usually 35–134 d. Generally, each adult lays 94–279 eggs with a maximum of 426 eggs [13,14,15].

Presently, *A. agrestis* is controlled mainly through chemical agents. However, the cryptic habit of slugs dwelling in the soil and their resistance to chemical control measures resulting from the extensive and widespread use of chemical agents have led to the decreasing effectiveness of chemical control. In addition, chemical control leads to pesticide residues, which are harmful to the ecosystem [16,17,18,19]. *A. agrestis* can also be controlled by agronomic and physical means. However, this incurs high costs and is neither efficient nor precise. Therefore, to achieve the sustainable management and safe control of slugs, biological control carries great potential, and relevant research is needed.

Biological control with insects is performed in integrated pest management, which is an environmentally friendly approach that emphasizes natural control, safety, and efficiency [20]. The natural enemies of slugs include various insect species (e.g., ground beetles, rove beetles, fireflies, and ants), nematodes, and several myriapods [3,21,22]. Carabid beetles, many of which are prominent gastropod-feeding insects, are beneficial organisms in agroecosystems because they are potential natural control agents against slugs [23,24,25]. Therefore, they could be used as an alternative to commercial molluscicides with the prospect of broad application [25]. 

The carabid beetle, *Carabus elysii* Thomson, is one of the more common beetles and is widely distributed in central, eastern, and southern China [26,27,28]. The adult beetle is 28–32 mm in length and 9–11 mm in width, and the body length of adult females is larger than that of adult males. The generation cycle is long, with only one generation a year [26,27]. The beetle is a nocturnal feeder and often hides in the soil between crevices, dead leaves, and weeds due to their aversion to light. The carabid species prey on the larvae of Lepidoptera, slugs, snails, and other small mollusks through adults and larvae and thus have potential for controlling slugs [26,29]. Studies on *C. elysii* have focused mainly on its morphology and distribution [30,31,32]. To our knowledge, few reports regarding its predation function exist. Investigating its predation of slugs is therefore vital for understanding this species’ potential for biological pest control. In this study, we investigated the predatory functional response, searching efficiency, and interference effects of *C. elysii* regarding predation on the terrestrial slug *A. agrestis* and discussed the potential for its use in the biological control of slugs.

## 2. Materials and Methods

### 2.1. Slugs and Beetles

*A.agrestis* individuals were collected from vegetable fields of the Fuguo Farming Cooperative, Yuqing County, Zunyi City, Guizhou Province (107. 47′ E, 27. 40′ N), and reared on *Lactuca sativa* Linnaeus in the laboratory to facilitate reproduction. According to an established method used for *Pomacea canaliculata* (Lamarck) [33], the slugs were categorized into three sizes on the basis of their body length (L) when straightened: juvenile (5 mm ≤ L < 15 mm), immature (15 mm ≤ L < 25 mm), and adult (25 mm ≤ L < 40 mm). The juvenile, immature, and adult slugs that exhibited consistent reproduction in the same generation were used as experimental prey.

*C.elysii* individuals were collected from Yelang National Forest Park, Hezhang County, Bijie City, Guizhou Province (104. 64′ E, 27. 08′ N). They were fed on *Tenebrio molitor* Linnaeus. Female and male adults with consistent reproduction in the same generation were used for the experiments.

Experiments were conducted in climate-controlled chambers (RXZ, Ningbo Jiangnan Instrument Factory, Ningbo, Zhejiang, China) at 25 °C ± 1 °C, 75% ± 5% relative humidity and L:D = 14:10 h photoperiod.

### 2.2. Functional Response

The predatory functional responses of female and male adults of *C. elysii* to *A. agrestis* larvae, immatures, and adults were studied separately. All experiments were conducted in square rearing containers (21 cm length × 14.3 cm width × 6.8 cm height). Twenty holes (1 cm diameter) in the cover ensured sufficient ventilation. Inside each container, an *L.* *sativa* leaf disk (9 cm length × 15 cm width) was placed upside down on wet filter paper to retain the leaf’s freshness. The prey densities of *A. agrestis* were 10, 20, 30, 40, and 50 individuals per container for larvae, and 5, 10, 15, 20, and 25 individuals per container for immatures and adults. All slugs and all beetles were starved for 24 h prior to the experiment. The predators were introduced individually to the containers. After 24 h exposure, the beetles were removed, and the number of residual preys was recorded. Each prey density was replicated 10 times.

The predatory functional response was calculated using a Holling type II [34] formula for fitting:

*N_a_* = *a’TN*/(1 + *a’T_h_N*) (1)

where *N* is the prey density, *N_a_* is the number of consumed preys, *T* is the time available for the predator to discover the prey (T = 24 h), *a’* is the instantaneous attack rate, and *T_h_* is the time required by the predator to process one individual (handling time). A nonlinear least-squares method was used to estimate the parameters *a’* and *T_h_*. The starting values of *a’* and *T_h_* needed by the NLR procedure were found using the linear regression of 1/*N_a_* against 1/*N*. The resultant y-intercept is the initial estimate of *T_h_*, and the reciprocal of the regression coefficient is an estimate of *a’*.

### 2.3. Searching Efficiency

According to the results of the parameters *a’* and *T_h_* obtained in Section 2.2, the searching efficiency (*S*) of *C. elysii* preying on *A. agrestis* was calculated according to the formula:

*S* = *a’*/(1 +*a’T_h_ N*) (2)

where *S* represents the finding effect, *a’*, *T_h_*, and *N* have the same meaning as in Section 2.2 [35].

### 2.4. Intraspecific Competition

The interference effects of female and male adults of *C. elysii* on larvae, immature and adult *A. agrestis*, were studied separately. The densities of predators per container were 1, 3, 5, and 7 female or male *C. elysii*. Female or male predators were introduced to each container containing 100 slugs. After 24 h of exposure, the predators were removed, and the number of residual preys was recorded. Each predator density treatment had five replicates.

The Hassell model equation was used as

*E* = *QP^−m^* (3)

where *E* is the mean predation rate per predator, *Q* is the search constant, *P* is the predator density, and *m* is the disturbance coefficient [36]. The values of *Q* and m were obtained from nonlinear regression models—the power exponential regressing of *E* and *P*. 

### 2.5. Data Analysis

The instantaneous attack rate (*a’*), handling time (*T_h_*), searching constant (*Q*), and disturbance coefficient (*m*) of female and male beetles preying on various sizes slugs were found through one-way analysis of variance (ANOVA) with post hoc comparisons using the LSD test, (*p* < 0.05). Data analysis was performed using Excel software (Microsoft Excel 2016, Microsoft, Redmond, WA, USA) and SPSS software (SPSS, version 21.0, Chicago, IL, USA). The goodness of fit of each equation was tested by a chi-square (*x*^2^) test.

## 3. Results and Analysis

### 3.1. Functional Response 

**Figure 1:**
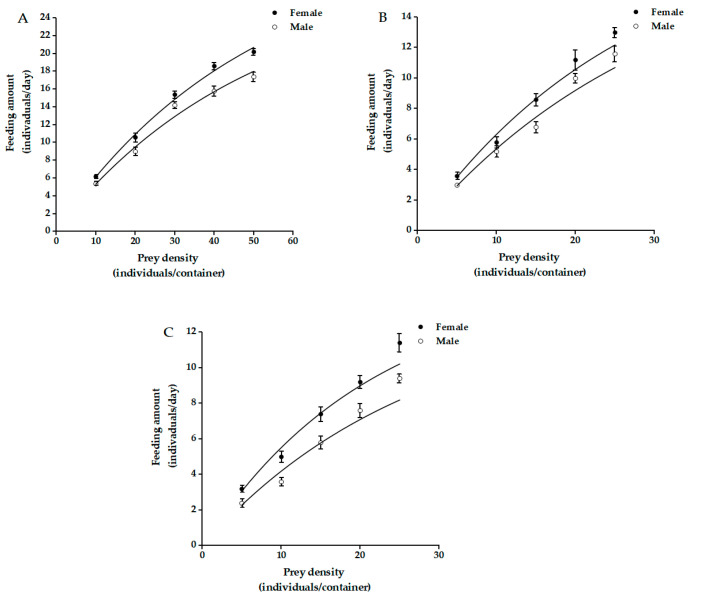
Fitting curve of predation function responses of *C. elysii* adults to juvenile (**A**), immatures (**B**), and adults (**C**) of *A. agrestis*.

The *A. agrestis* predation capacity of *C. elysii* adults increased gradually with an increase in prey density (Figure 1). However, the predation growth rates (decreasing rate of increase in the feeding rate) of *C. elysii* adults preying on *A. agrestis* decreased with increasing prey density (Figure 1), which is an indicator of type II functional response. The values of the correlation coefficient *R*^2^ of the functional response (0.9148–0.9871) were greater than 0.9 (Table 1), revealing a significant correlation between the observed and the predicted predated-prey number. The values *x*^2^ =0.0623–0.2090 with the chi-square goodness-of-fit tests concerning predicted and observed predation were substantially lower than the threshold of *x*^2^_(0.05, 4)_ = 9.49. This indicates that the fitted Holling type II disc equation reflected the predation by *C. elysii*.

Using Holling’s disc equation, the coefficients of *a’* (instantaneous attack rate) and *T_h_* (handling time) were estimated for *C. elysii* males and females (Table 1). There was no significant difference in *a’* between female and male *C. elysii* feeding on juvenile and immature *A. agrestis*, and female beetles consumed significantly more adults than the male beetles. Female beetles exhibited significantly higher Th for slug adults than juveniles and immatures, and no significant difference in case of male beetles. The maximum daily prey consumption by female adults was 35.4895 (juvenile), 25.0841 (immature), and 17.1168 (adult), and the prey consumption by male adults was 26.9042 (juvenile), 20.2789 (immature), and 11.6290 (adult). 

### 3.2. Searching Efficiency 

**Figure 2:**
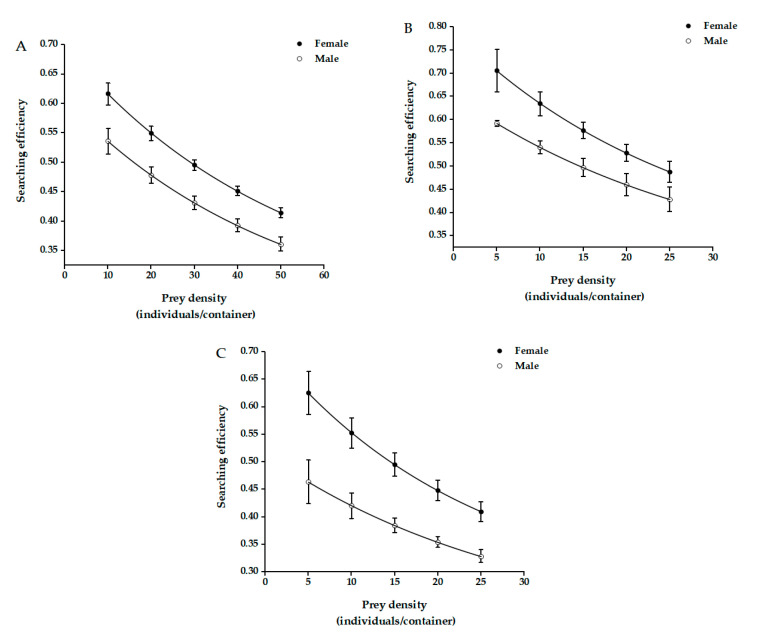
Searching efficiency of *C.* *elysii* adults for juvenile (**A**), immature (**B**), and adult (**C**) *A.* *agrestis* individuals.

The searching efficiency of both female and male *C. elysii* adults regarding distinct categories of *A.* *agrestis* decreased with increasing prey density. The searching efficiency of female *C. elysii* adults regarding *A. agrestis* was always higher than that of male adults at identical ages and densities of *A. agrestis* (Figure 2).

### 3.3. Intraspecific Competition 

**Figure 3:**
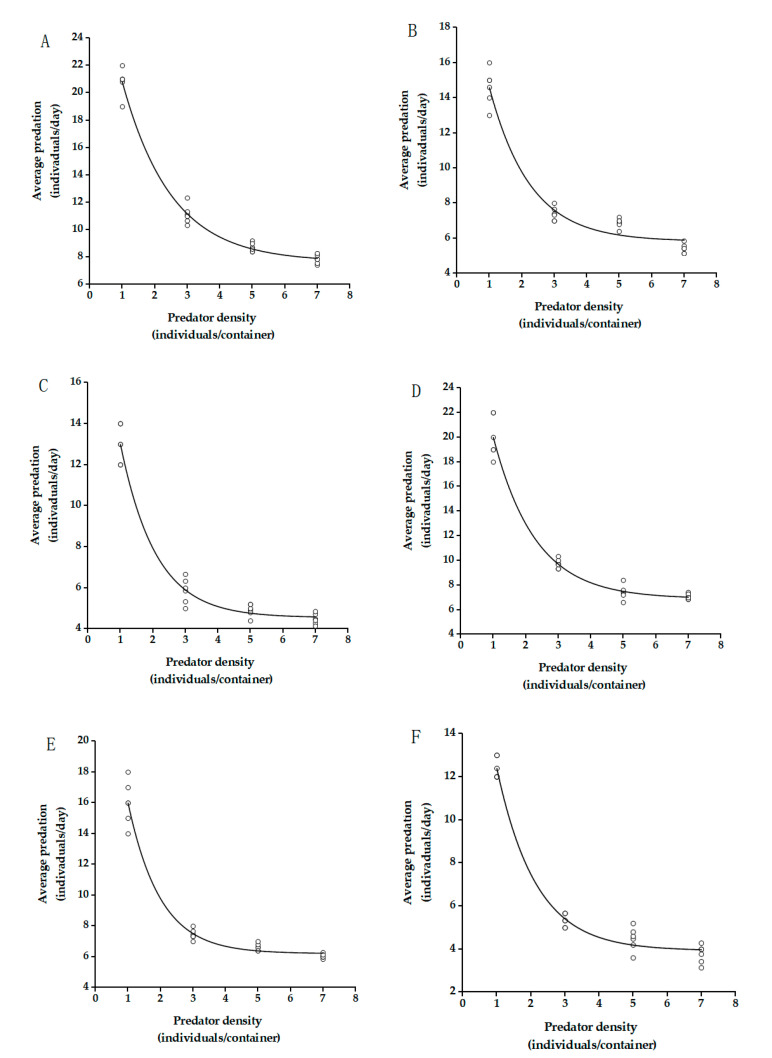
Interspecific disturbance responses of male and female adults of *C. elysii* preying on *A. agrestis*; interspecific interference response of female adults to juvenile (**A**), immature (**B**), and adult (**C**) *A. agrestis*; Interspecific interference response of male adults to juvenile (**D**), immature (**E**), and adult (**F**) *A. agrestis*. Each data point represents the average predation of *C. elysii* adults at different densities. Curves were fitted using the intraspecific competition equation (Equation (3)).

With increasing density of *C. elysii*, its predation on *A. agrestis* increased. However, the predation rate per adult gradually decreased (Figure 3). The equation *E* = *QP*^−*m*^ of the Hassell model was used to fit the predator density. The values of the correlation coefficient *R*^2^ of the functional response were greater than 0.99, revealing that the predation rate of *C. elysii* was significantly correlated with its density and predator density interfered with its predation on slugs(Table 2). In other words, an increase in the density of predators led to an increase in the interference effect between predator individuals, which caused a decrease in the overall predation rate. The *Q* and *m* values of both male and female adults were significantly different for different sizes of slugs.

## 4. Discussion

**Figure A1:**
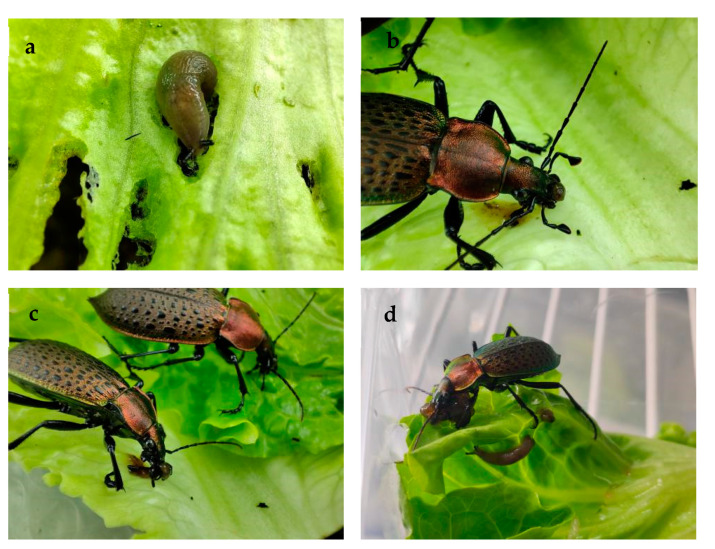
*A. agrestis* damaging lettuce (**a**); female adult of *C. elysii* feeding on *A. agrestis* (**b**); male adult of *C. elysii* attacking *A. agrestis* (**c**,**d**).

Studying predation by natural enemies is important for determining the potential of certain species to control specific target pests [37]. The carabid beetles *Harpalus rufipes* (De Geer), *Pterostichus melanarius* (Illiger), *Abax parallelepipedus* (Piller and Mitterpacher), *Poecilus cupreus* Linnaeus, *Carabus nemoralis* Müller, and *Pterostichus niger* (Schaller) have been reported as prominent predators of slugs (e.g., *Deroceras reticulatum* (Müller), *Arion lusitanicus* Mabille) [38,39,40,41]. The effectiveness of natural enemies against pests is often evaluated by a predator–prey model using the Holling functional response, in which the maximum daily prey consumption (*a’*/*T_h_*) is a vital parameter that shows the predation efficacy of natural enemies against the prey. A greater *a’*/*T_h_* value expresses a greater control of natural enemies against pests [42]. In this study, the functional responses of female and male *C. elysii* to *A. agrestis* followed the Holling type II model. Female and male adults of the ground beetle are functionally capable of preying on different-sized (i.e., juvenile, immature, and adult) terrestrial slugs (Figure A1). The predation levels of the prey decreased with slug development, and the juveniles were the most affected prey. Previous studies have suggested that other carabid beetle species have a higher predation preference and capacity for small slugs. El-Danasoury et al. (2017) and El-Danasoury and Iglesias-Piñeiro (2018) reported that *H. rufipes* showed remarkable predation on the eggs and larvae of *D. reticulatum* under indoor conditions. The beetles’ ability to prey on small juveniles (≤5.0 mg) was greater than their ability to prey on medium-sized juvenile slugs (10–20 mg) and large juveniles (50–60 mg) [43,44]. McKemey et al. (2001) also found that under indoor conditions *P. melanarius* only fed on *D. reticulatum* larvae (<40 mg) and eggs and rarely fed on slugs over 40 mg [39,45]. The medium-sized slugs of *D. reticulatum* could be attacked by *P. melanarius* in the absence of alternative prey, but large slugs were not [38]. This situation is similar to that observed in the predation of slugs by *C. nemoralis*, *C. violaceus*, *Pterostichus*. *madidus* (Fabricius), *A. parallelepipedus*, etc. [40,41,46,47]. The body size of slugs has a greater effect on the predatory abilities of their natural enemies, which may be related to the defense mechanism of slugs [43]. Hanlon et al. (2008) observed that slugs secrete large amounts of calcium to increase the mucus’s viscosity to defend against natural enemies when they are exposed to damage [48,49]. Their ability to secrete mucus and defend against natural enemies is gradually strengthened as their defense mechanism progressively improves as they increase in body size.

Our study found that the maximum daily predation of slugs by female predators was more than that of the male adults, indicating that female predators have a greater predation ability on slugs. Oberholzer and Frank (2003) found that there was little distinctiveness between the predation rates of male and female adults of *P. melanarius* on *D. reticulatum* and no gender differences in *P. cupreus* [38]. No differences in the predation of *P. melanarius* on different slugs were found between the sexes either [50]. However, Symondson (1989) found that the female beetles ate more than the males, although their predation rates were similar when *A. parallelepipedus* fed on *D. reticulatum* [51]. Other predatory insects, such as *Eocanthecona furcellata* (Wolff), *Arma chinensis* (Fallou), and *Orius similis* Zheng on *Spodoptera frugiperda* (Smith) found that adult females consumed more prey than adult males [52,53,54]. All of these natural enemies showed larger body sizes in females than in males, which may be a reason for the difference in predation between males and females. Furthermore, females usually need to store more energy to supply their subsequent need to lay eggs.

The searching behavior of predators seeking to consume prey is called searching efficiency [35]. In our experiments, it was found that female adult skeptic beetles were more efficient in searching for slugs than male adults. The natural enemies mainly rely on the receptors on olfactory, auditory, tactile, and visual to search for prey, especially on the antennae, which are the main receptor organs of insects [55]. Some studies have shown that there was no significant difference in the morphological distribution of receptors on the antennae of female and male *C. elysii*, regardless of the fact that the total number of receptors in female adults was significantly higher than in male adults [31,32]. It was speculated that this may be the reason for the difference in the search effect between male and female adults.

Our experiments indicated that the density of the carabid beetle itself had a significant effect on predation on the same density of ground slugs. With the increase in density, the total predation gradually increased, but the average predation and predation rate gradually decreased. Mou et al. (2005) showed that total predation increased with predator density with the consumption of three species of lepidopteran larvae, *Lampronadata* *cristata* (Butler), *Phalera assimilis* (Bremer et Grey) and *Hypocala subsatura* (Guenee) by *Calosoma maximoviczi* Morowitzi [56]. However, the high density increased the mutual interference between individuals, decreasing the predation rate. Similarly, this phenomenon was found in the predation of other common predatory natural enemies, such as *Propylea japonica* (Thunberg), *Menochilus sexmaculata* (Fabricius), *Orius sauteri* (Poppius), etc. [37,57,58].

## 5. Conclusions

In this study, the results of the predation ability of female and male *C. elysii* on different sizes of *A. agrestis* under indoor conditions showed that the carabid beetle has strong potential to prevent and control the terrestrial slug. Future research should be conducted on the predation ability of larvae *C. elysii*, the biological characteristics of *C. elysii*, and the effects of different ecological factors on predation. Simultaneously, the actual occurrence features of slugs in the field, the influence of environmental factors, etc., should be fully considered for the systematic evaluation of the predation ability and pest control effect of the carabid beetle on terrestrial slugs. The release pattern, release density, and release time conditions of natural enemies in field conditions were clarified to lay the theoretical foundation for green ecological control of slugs.

## Figures and Tables

**Table 1:** Functional responses of *C. elysii* adults to distinct sizes of *A. agrestis*.

Prey Ages	Predator Sexes	*R* ^2^	*a’*	*T_h_*	Maximum Daily Prey Consumption
Juvenile	Female	0.9871	0.7027 ± 0.0313 ab	0.0198 ± 0.0014 b	35.4895
	Male	0.9762	0.6107 ± 0.0364 bc	0.0227 ± 0.0030 ab	26.9042
Immature	Female	0.9642	0.7952 ± 0.0800 a	0.0317 ± 0.0090 ab	25.0841
	Male	0.9691	0.6550 ± 0.0073 abc	0.0323 ± 0.0065 ab	20.2789
Adult	Female	0.9717	0.7206 ± 0.0612 ab	0.0421 ± 0.0054 a	17.1168
	Male	0.9148	0.5175 ± 0.0664 c	0.0445 ± 0.0115 a	11.6290

*R*^2^: the coefficient of determination estimated by fitting Holling II disc equations; *a’*: attack rate (d^–1^). *T_h_*: handling time (d); *a’* and *T_h_* were estimated via nonlinear least-squares regression. Mean ± SE of *a’* and *T_h_* values with different lowercase letters in the same column indicate significant differences (*p* < 0.05, LSD test).

**Table 2:** Interference response coefficients of *C.* *elysii* density to distinct sizes of *A.* *agrestis*.

Prey Ages	Predator Ages	*R* ^2^	*Q*	*m*
Juvenile	Female	0.9983	0.5008 ± 0.0036 a	1.5112 ± 0.0111 ab
	Male	0.9956	0.4886 ± 0.0086 a	1.5561 ± 0.0307 ab
Immature	Female	0.9958	0.4273 ± 0.0054 b	1.4913 ± 0.0230 c
	Male	0.9931	0.4392 ± 0.0066 b	1.5084 ± 0.0238 bc
Adult	Female	0.9918	0.4023 ± 0.0038 c	1.5598 ± 0.0177 bc
	Male	0.9915	0.3953 ± 0.0040 c	1.6134 ± 0.0182 a

*E* is the mean predation rate per predator; *R*^2^ is the coefficient of determination estimated by fitting interference response equations; *Q* is the searching constant; *m* is the disturbance coefficient. Mean ± SE of with different lowercase letters in the same column indicate significant differences (*p* < 0.05, LSD test).

## Data Availability

The data represented in this study are available on request from the corresponding author.
